# The end of zero-COVID-19 policy is not the end of COVID-19 for China

**DOI:** 10.1016/j.lanwpc.2023.100702

**Published:** 2023-01-31

**Authors:** The Lancet Regional Health – Western Pacific

China officially ended its zero-COVID-19 policy on Jan 8, 2023. The management of COVID-19 is now downgraded from Class A to Class B in accordance with the country's law on infectious diseases prevention and treatment. There is no more centralised quarantine, close contact tracing, or mass nucleic acid testing. China now enters a new phase of the COVID-19 response.

The end of the zero-COVID-19 policy reminds us to reflect on its gains and losses. Beyond all doubts, the dynamic zero-COVID-19 policy has protected the most vulnerable populations from five global COVID-19 waves and avoided widespread infections with the original strain and the delta variant. Its border closure policy blocked, or at least postponed, the entry of variants from abroad. It also won precious time for the whole society to be better prepared for high infection rates in the population. Nevertheless, the zero-COVID-19 policy also had some disadvantages: a low number of infections among the population resulted in a low level of natural immunity compared with other countries; a lack of urgency to get vaccinated fostered vaccine hesitancy; stringent border measures halted international cooperation; policies such as frequent mass testing and quarantine treatment have disrupted people's life and work, as well as increased the financial burden on government; and other diseases have been neglected due to an excessive focus on COVID-19. Moreover, the abrupt lockdowns in several cities last year in response to outbreaks of the omicron variant exposed the adverse effects of the stringent zero-COVID-19 policy, such as family separations, food supply shortages, and impaired access to medical care.

In this new phase of the pandemic, China is facing unprecedented challenges, as well as opportunities for strengthening its public health systems. The domestic impact of the shift from the strict policy is immediate—people are going through an abrupt outbreak of COVID-19 before life returns to normal. Despite the small number of reported cases, there are signs of a sharp rise in recent infections nationwide and the actual number could be much larger than the official data due to the change in testing requirements. According to a recent preprint, the infection incidence might have already peaked in Beijing on Dec 11, 2022. A model developed by the Institute for Health Metrics and Evaluation projects over 300,000 deaths in China in the coming months, and more than a million over the course of 2023 after the policy shift. In fact, hospitals are already overwhelmed due to the increased numbers of people with fever attending clinics, and anti-fever drugs were out of stock for weeks. Large numbers of medical staff are infected while still going to work sick, staying on the frontline, and coping with the surge in patients with fever. Long-term, China will face looming challenges such as the health burden of post-COVID-19 condition (also known as long COVID).

Hopefully, the following efforts might help to address these challenges. A modelling study posted on medRxiv suggests the combined effect of vaccination (≥85% coverage of the fourth dose as a booster) and antiviral treatment (≥60% coverage) could substantially reduce COVID-19 morbidity and mortality as China transitions from dynamic-zero to normality. China is on the way to achieve these targets. The government is speeding up the booster vaccinations, especially for the older population. It has also started administering a fourth COVID-19 vaccine dose. Antiviral drugs are now allocated at hospitals and primary medical care centres in major cities such as Beijing, Shanghai, and Guangzhou, with the daily output increased by more than four times in late December compared with early December. China has always wanted to strengthen its primary health-care system. Now, by promoting a tiered diagnosis and treatment system, expanding internet-based medical services, and allocating medical resources to the primary health-care centres, there is a good opportunity to let people get used to and build trust in primary health-care systems such as community hospitals and online medical services, instead of the over-reliance on top-grade hospitals.

From a global perspective, China's reopening has been welcome, but has also led to some concerns. Several countries are setting inbound travel restrictions targeting only Chinese travellers, owing to concerns about the sufficiency and adequacy of Chinese data on COVID-19 infections. China can do better in co-operating with the global community to overcome the pandemic by sharing information in a more timely, open, and transparent manner. Clinical data on the current COVID-19 situation, medical treatment, and vaccination, as well as research findings of the transmissibility, severity, vaccine escape of variants and antiviral resistance, and vaccine effectiveness should be made available. Data reporting should also be as consistent as possible, and in this respect, China is encouraged to change its COVID-19 death definition in line with the WHO recommendation to accurately calculate excess deaths. With the end of the zero-COVID-19 policy in China, nationwide coordination is more important than ever amid the continuing global fight against the pandemic.

